# Inflammation-based Scores in Patients With Pheochromocytoma

**DOI:** 10.1210/clinem/dgae284

**Published:** 2024-04-24

**Authors:** Chiara Parazzoli, Alessandro Prete, Vittoria Favero, Carmen Aresta, Valentina Pucino, John Ayuk, Miriam Asia, Yasir S Elhassan, Iacopo Chiodini, Cristina L Ronchi

**Affiliations:** Department of Biotechnology and Translational Medicine, University of Milan, 20100 Milan, Italy; Institute of Metabolism and Systems Research, University of Birmingham, Birmingham B15 2TT, United Kingdom; Institute of Metabolism and Systems Research, University of Birmingham, Birmingham B15 2TT, United Kingdom; Centre for Endocrinology, Diabetes and Metabolism, Birmingham Health Partners, Birmingham B15 2TT, UK; Department of Endocrinology, Queen Elizabeth Hospital Birmingham, Birmingham B15 2GW, UK; National Institute for Health Research Birmingham Biomedical Research Centre, University of Birmingham and University Hospitals Birmingham NHS Foundation Trust, Birmingham B15 2TH, UK; Department of Biotechnology and Translational Medicine, University of Milan, 20100 Milan, Italy; Department of Endocrine and Metabolic Diseases, IRCCS, Istituto Auxologico Italiano, 20100 Milan, Italy; Kennedy Institute of Rheumatology, University of Oxford, Oxford OX3 7FY, UK; Institute of Inflammation and Ageing, University of Birmingham, Birmingham B15 2TT, UK; Department of Endocrinology, Queen Elizabeth Hospital Birmingham, Birmingham B15 2GW, UK; Department of Endocrinology, Queen Elizabeth Hospital Birmingham, Birmingham B15 2GW, UK; Institute of Metabolism and Systems Research, University of Birmingham, Birmingham B15 2TT, United Kingdom; Centre for Endocrinology, Diabetes and Metabolism, Birmingham Health Partners, Birmingham B15 2TT, UK; Department of Endocrinology, Queen Elizabeth Hospital Birmingham, Birmingham B15 2GW, UK; Department of Biotechnology and Translational Medicine, University of Milan, 20100 Milan, Italy; Unit of Endocrinology, ASST Grande Ospedale Metropolitano Niguarda, 20162 Milan, Italy; Institute of Metabolism and Systems Research, University of Birmingham, Birmingham B15 2TT, United Kingdom; Centre for Endocrinology, Diabetes and Metabolism, Birmingham Health Partners, Birmingham B15 2TT, UK; Department of Endocrinology, Queen Elizabeth Hospital Birmingham, Birmingham B15 2GW, UK

**Keywords:** inflammation scores, pheochromocytoma, catecholamines, metanephrines, alfa-blockade

## Abstract

**Background:**

Pheochromocytoma is associated with systemic inflammation but the underlying mechanisms are unclear. Therefore, we investigated the relationship between plasma metanephrine levels and hematological parameters—as a surrogate of inflammation—in patients with pheochromocytoma and the influence of preoperative α-blockade treatment.

**Design and Methods:**

We retrospectively studied 68 patients with pheochromocytoma who underwent adrenalectomy (median age, 53 years; 64.7% females) and 2 control groups matched for age, sex, and body mass index: 68 patients with nonfunctioning adrenocortical tumors and 53 with essential hypertension. The complete blood count and several inflammation-based scores (neutrophil-to-lymphocyte ratio [NLR], platelet-to-lymphocyte ratio [PLR], lymphocyte-to-monocyte ratio [LMR], systemic-immune-inflammation index [SII], prognostic-nutrition index) were assessed in all patients and, in a subset of pheochromocytomas, after adrenalectomy (n = 26) and before and after preoperative α-blockade treatment (n = 29).

**Results:**

A higher inflammatory state, as indicated by both complete blood count and inflammation-based scores, was observed in patients with pheochromocytoma compared with nonfunctioning adrenocortical tumors and essential hypertension. Plasma metanephrine levels showed a positive correlation with NLR (*r* = 0.4631), PLR (*r* = 0.3174), and SII (*r* = 0.3709) and a negative correlation with LMR (*r* = 0.4368) and prognostic-nutrition index (*r* = 0.3741), even after adjustment for age, sex, ethnicity, body mass index, and tumor size (except for PLR). After adrenalectomy, we observed a reduction in NLR (*P* = .001), PLR (*P* = .003), and SII (*P* = .004) and a concomitant increase in LMR (*P* = .0002). Similarly, α-blockade treatment led to a reduction in NLR (*P* = .007) and SII (*P* = .03).

**Conclusion:**

Inflammation-based scores in patients with pheochromocytoma showed pro-inflammatory changes that correlated with plasma metanephrine levels and are ameliorated by adrenalectomy and α-blockade.

Pheochromocytomas are rare neuroendocrine tumors arising from the chromaffin cells of the adrenal medulla that typically secrete excessive amounts of catecholamines (1). Chronic exposure to high levels of catecholamines is responsible for most of the clinical manifestations of pheochromocytoma, including the classic triad of headache, palpitations, and profuse sweating, as well as significant hemodynamic and metabolic changes.

Beyond their well-known effects on the cardiovascular system (2, 3) and metabolism (4, 5), catecholamines also influence the immune system. Previous studies have shown that catecholamines directly modulate innate immune cell function in vitro and in vivo (6-9) and regulate the production of pro-inflammatory cytokines (10). The effects of catecholamines on the immune system are mediated by the adrenergic receptors expressed by immune cells. The β2-adrenoceptor is thought to be most involved in inflammatory processes, but increasing evidence suggests the role of other adrenergic receptors, particularly the α1 subtype (11-13). So far, some evidence suggests that patients with pheochromocytoma may have an increase in several inflammatory markers, which recovers after the tumor removal (9, 14, 15). Interestingly, patients with pheochromocytomas and paragangliomas and an increased inflammatory state have been suggested to have a reduced survival (16).

Recently, several inflammation-based scores have been proposed as potential markers of systemic inflammation in several diseases, such as ischemic heart disease, stroke, and cancer (17-20). The increasing interest in these markers is due to their recognized prognostic value as well as their cost-effectiveness, wide availability, and practicality. The combination of common serum-based parameters, such as complete blood count (CBC) and acute-phase proteins, can predict acute and chronic inflammation. An increase in the neutrophil-to-lymphocyte ratio (NLR), platelet-to-lymphocyte ratio (PLR), and systemic immuno-inflammation index (SII; the product of platelet count and NLR), and a decrease in the lymphocyte-to-monocyte ratio (LMR) and prognostic nutrition index (PNI; that consider serum albumin and the absolute lymphocyte count) reflect ineffective immune surveillance or an increased inflammatory state (21).

Scarce data are available about the relationship between the inflammation-based scores and catecholamines secretion in pheochromocytomas and, in particular, the effect of the normalization of catecholamine secretion. Moreover, the possible role of α-blockers in modulating these parameters is unknown.

The aim of this study was, therefore, to evaluate: (1) the levels of several inflammation-based scores in patients with pheochromocytoma before and after adrenalectomy and their relationship to clinical characteristics and catecholamine levels and (2) the possible impact of α-blockers on the inflammation-based scores.

## Subjects and Methods

### Patient Cohort

We performed a retrospective electronic clinical records review of 68 patients with pheochromocytoma, 68 patients with nonfunctioning adrenal tumors (NFAT), and 53 patients with essential hypertension (EAH).

Patients with pheochromocytoma were diagnosed between January 2001 and April 2023 and followed up in the Adrenal Tumor Service at the Queen Elizabeth Hospital Birmingham (UK). Only patients with pheochromocytomas who underwent surgery with subsequent normalization of catecholamine levels and with available clinical and biochemical data including CBC before and after adrenalectomy were included. In accordance with current guidelines (1), the diagnosis of pheochromocytoma was confirmed histologically or based on the combined presence of increased plasma metanephrines and detection of an indeterminate adrenal mass on imaging. If the exact values of metanephrines and normetanephrines were not available because they were initially diagnosed in another center, the pheochromocytomas were considered hypersecretive based on the information in the referral letter. Patients with paragangliomas or metastatic pheochromocytomas were excluded. Patients with conditions that could significantly affect the CBC, such as infections, hematological diseases, severe cardiomyopathy, active malignancies, active autoimmune diseases, and treatment with oral glucocorticoids or other immunomodulatory drugs, were also excluded (Supplementary Fig. S1) (22).

Patients with NFAT or EAH matched for age, sex, and body mass index (BMI) to the cohort of pheochromocytoma; those with available CBC were used as control groups (Supplementary Table S1) (22).

NFAT were defined by the presence of adrenocortical adenomas and cortisol values after 1-mg overnight dexamethasone suppression test <50 nmol/L (1.8 µg/dL) (23). Patients with NFAT were followed at the Adrenal Tumor Service of the Queen Elizabeth Hospital, Birmingham, UK (diagnosed between January 2001 and April 2023).

EAH were defined according to international guidelines (24), and without conditions associated with increased activity of the hypothalamic-pituitary-adrenal axis, including diabetes mellitus type 2 (T2DM). Patients with any of these conditions that could affect the CBC were excluded. Patients with EAH were followed at the Hypertension Centre of the Istituto Auxologico Italiano, Milan, Italy, between June 2019 and April 2023.

### Study Design

Data were collected at the time of tumor diagnosis (baseline) and at 3 different time points after adrenalectomy: (1) at the last day of hospital admission (immediately postsurgery, n = 68, median time 3 days, interquartile range [IQR] 2-5); (2) at least 1 month after and within 1 year of surgery (short-term follow-up, n = 18, median time 5 months, IQR 1. 8-7.9); and (3) at least 1 year after surgery (long-term follow-up, n = 15, median time 39.6 months, IQR 26.4-55.2). In patients with bilateral metachronous tumors, defined as pheochromocytomas developing in a contralateral side after at least 6 months of the initial tumor (25), data before and after second surgery were considered. Control patients were assessed only once.

This study has been conducted in accordance with the Declaration of Helsinki. Institutional review board approval for retrospective data review from patients with pheochromocytoma undergoing routine clinical care was obtained from the University Hospital Birmingham NHS Foundation Trust (audit reference CARMS-18152). Ethical approval has been obtained for study research by both local institutions (NHS Health Research Authority—Prime-Act study IRAS 261291, RG 19-028, and Istituto Auxologico Italiano of Milan Code 2019_01_29_06).

### Data Collection

Demographic and clinical data were collected for all patients at the time of diagnosis, including the presence of adrenergic symptoms (ie, palpitations, sweating, tremors, anxiety) as well as the cardiometabolic comorbidities typically associated with catecholamine excess, such as hypertension, cardio-cerebrovascular events (CVE), T2DM, and obesity (BMI >30 kg/m^2^). Additionally, details of antihypertensive treatment, including α-blockade, were collected in patients with pheochromocytoma. The immediate preoperative α-blocker dose was considered in the analysis. The biochemical evaluation included the determination of plasma metanephrines and normetanephrine (data available in 59 and 62 patients, respectively), as well as CBC, serum albumin levels, and C-reactive protein (CRP) levels when available (n = 38, all measurements with values ≤10 mg/L). Plasma metanephrines and normetanephrine were measured by liquid chromatography-tandem mass spectrometry using the Chromsystems MassChrom Free Metanephrines in Plasma commercial kit. Plasma metanephrine and normetanephrine at the time of CBC sampling were used for the analysis.

Inflammation-based scores were calculated from serum albumin and CBC (Supplementary Table S2) (17, 21, 22). NLR and PLR were calculated by dividing the absolute neutrophil or platelet counts, respectively, by the lymphocyte count. LMR is obtained by dividing the absolute lymphocyte count by the monocyte count, whereas the SII is the product of the absolute platelet count and NLR. The PNI reflects not only the inflammatory status but also the nutritional status of the patient and is obtained by multiplying serum albumin by 5 times the absolute lymphocyte count.

Radiological features of the adrenal mass, such as maximum diameter and side of the lesion, were also collected. For bilateral adrenal tumors, the diameter of the largest mass was considered.

Two different histopathological scores (Pheochromocytoma of the Adrenal Gland Scaled Score [PASS] and Grading system for Adrenal Pheochromocytoma and Paraganglioma) were recorded (26) and details about postoperative complications and duration of hospital admission were specified. In addition, data about genetic screening were collected. Genetic testing for germline variants that predispose to pheochromocytoma was offered to all patients who met the national eligibility criteria (27). A targeted next-generation sequencing gene panel was used for genetic testing, evaluating coding regions in *FH, MAX, MEN1, RET, SDHAF2, SDHA, SDHB, SDHC, SDHD, TMEM127,* and *VHL. NF1* was tested only in the presence of clinical features of neurofibromatosis type 1. Genetic data were not available for 26 patients because (1) they did not meet eligibility criteria for genetic testing, (2) they did not provide consent, or (3) test results were pending at the time the study was conducted.

### Statistical Analysis

Descriptive statistics were expressed as numbers and percentages for categorical variables and as median and IQR for continuous variables. Comparisons between patients with pheochromocytoma, NFAT, and EAH were performed using the Mann-Whitney *U* test and Kruskal-Wallis test followed by the Dunn post hoc test. Analysis of the paired continuous values (data pre- and postoperative as well as before and after α-blockade treatment) was performed using the Wilcoxon test. Spearman correlation analysis was performed to evaluate the relationship between metanephrines/normetanephrine and inflammatory parameters in patients with pheochromocytoma before surgery. Uni- and multivariate linear regression were performed to confirm the association found between the metanephrine levels and inflammation-based scores and adjust for age, sex, ethnicity, BMI, and tumor size of pheochromocytoma. In this analysis, all continuous variables were transformed to the natural logarithm of their value.

A *P* value of <.05 was considered statistically significant. Statistical analysis was performed by GraphPad Prism version 9.

## Results

### Patient Characteristics at Baseline

A total of 68 patients with pheochromocytoma were included in the study. Clinical characteristics are shown in Table 1. The majority were females (n = 44, 64.7%) of Caucasian ethnicity (n = 46, 67.6%) with a median age at diagnosis of 53 years (IQR 41.3-69). Among patients who underwent genetic testing to assess the presence of germline mutations predisposing to pheochromocytoma, 33.3% were found to have genetic defects, most commonly in the *RET* gene (n = 6). The most common mode of presentation was detection during workup for an adrenal incidentaloma (n = 32, 47.1%), followed by adrenergic symptoms (n = 31, 45.6%) and detection during screening for a known underlying genetic susceptibility (n = 5, 7.4%). Regarding the associated cardiometabolic morbidities, most patients were hypertensive (n = 52, 76.5%), of which 35.3% (n = 24) were taking more than 1 antihypertensive medication; T2DM and obesity were present in 25% and 30.9%, respectively. The biochemical evaluation showed that most pheochromocytomas had hypersecretion of both metanephrine and normetanephrine (n = 40, 58.8%), and radiologically, they generally presented an indeterminate mass with a median tumor size of 4.9 cm (IQR 3.6-6.5).

**Table 1.** Characteristics of the 68 patients with pheochromocytoma included in the study

Parameter	Value
Median age (IQR), y	53 (41.3-69)
Gender, F/M (%F)	44/24 (64.7)
Ethnicity, n (%)	
Caucasian	46 (67.6)
Non-Caucasian	10 (14.7)
Unknown	12
Median BMI (IQR), kg/m^2^	26.7 (24-31.2)
Active smoker, n (%)	8 (11.8)
Unknown	18
Germline mutations*^a^*, n (%)	
Negative	28 (66.7)
Positive	14 (33.3)
*RET (MEN2A)*	6 (8.8)
*NF1*	3 (4.4)
Others*^b^*	3 (4.4)
*VHL*	2 (2.9)
Unknown*^c^*	26
Presentation, n (%)	
Incidentally discovered	32 (47.1)
Symptoms of pheochromocytoma	31 (45.6)
Screened for genetic susceptibility	5 (7.3)
Cardiovascular-metabolic comorbidities, n (%)	
CVE	13 (19.1)
HTN	52 (76.5)
T2DM	17 (25)
BMI >30 kg/m^2^	21 (30.9)
Catecholamine excess, n (%)	
Both MN and NMN	40 (58.8)
NMN	19 (27.9)
MN	7 (10.3)
Nonsecreting	2 (2.9)
Tumor laterality, n (%)	
Unilateral*^d^*	61 (89.7)
Bilateral*^e^*	7 (10.3)
MIBG scintigraphy avidity, n (%)	
High	51 (75)
Negative	4 (5.9)
Unknown	13
Median diameter (IQR), cm	4.9 (3.6-6.5)
Median Hounsfield Unit (IQR), n = 19	32 (29-41)
Type of surgery, n (%)	
Unilateral	61 (89.7)
Bilateral synchronous	4 (5.9)
Bilateral metachronous	3 (4.4)
Median PASS score (IQR), n = 61	5 (3-7.5)
Median GAPP score (IQR), n = 11	5 (4-6)
Postoperative complications*^f^*, n (%)	14 (20.6)

Categorical variables are reported as N (%); continuous variables are reported as median (IQR).

Abbreviations: BMI, body mass index; CVE, cardiovascular events; F, female; GAPP, Grading system for Adrenal Pheochromocytoma and Paraganglioma; HTN, hypertension, T2DM, type 2 diabetes mellitus; IQR interquartile range; M, male; MEN2A, Multiple Endocrine Neoplasia Type 2A; MIBG, meta-iodobenzylguanidine; MN, metanephrine; n, number; NF1, neurofibromatosis type 1; NMN, normetanephrine; PASS, Pheochromocytoma of the Adrenal Gland Scaled Score; VHL, Von Hippel Lindau.

^*a*^Genetic test for germline mutations was undertaken using next-generation sequencing of coding regions in *FH, MAX, MEN1, RET, SDHAF2, SDHA, SDHB, SDHC, SDHD, TMEM127,* and *VHL*; *NF1* was tested only in the presence of clinical features of neurofibromatosis type 1.

^*b*^Mutation in *MAX* gene in 2 patients and in *TMEM127* gene in 1 patient.

^*c*^Data are not available because patients have no eligibility criteria to genetic testing, did not provide consent, or genetic testing was still ongoing.

^*d*^Right-sided in 35 (51.5%) patients; left-sided in 29 (42.6%) patients.

^*e*^For bilateral tumors, the maximum diameter of the larger adrenal mass was considered.

^*f*^Complications occurring during hospital admission after surgery were hospital-acquired pneumonia in 7 patients, surgical wound infection in 2 patients, cardiac arrhythmia in 2 patients, and urinary tract infection, type 2 respiratory failure, and post-operative bleeding in 1 patient each.

There was no difference in inflammation-based scores between patients with pheochromocytoma with and without hypertension, T2DM, CVE, and between incidentally detected and symptomatic patients (Table 2). Furthermore, tumors with PASS ≥ 4 (potentially aggressive behavior) and those with lower PASS (<4) had similar inflammation-based scores; no significant gender differences were observed (Table 2). The presence of germline mutations did not influence the scores (Table 2), even when the specific genes were considered individually (data not shown). In contrast, inflammation-based scores differed based on ethnicity, BMI, and biochemical phenotype (Table 2). In particular, higher NLR and lower LMR levels were observed in Caucasian patients (*P* = .005 and *P* = .001, respectively) compared with subjects with other ethnicity; patients with obesity had lower NLR (*P* = .02), PLR (*P* = .0005), and SII (*P* = .0009) and higher LMR (*P* = .04) than those with BMI <30 kg/m^2^ (Table 2). Catecholamine levels were further analyzed in these 2 categories and significant differences in metanephrines were found between the ethnic groups. Caucasian patients had higher metanephrine levels than the other ethnic groups (2645 [906-5264] vs 366.5 [173.3-1799], *P* = .02), but the differences in NLR and LMR remained significant even after adjustment for plasma metanephrine levels and BMI (*P* = .02 and *P* = .002, respectively—data not shown). Although not significant, patients with lower BMI have higher catecholamine levels than obese patients (metanephrine 2155 [363-5200] vs 1073 [342.8-2225], *P* = .2; normetanephrine 8201 [2683-25,000] vs 5189 [1893-16,986], *P* = .6). Considering the biochemical phenotype, we found that pheochromocytomas secreting only normetanephrines had lower NLR values (*P* = .03) and higher LMR (*P* = .03) and PNI (*P* = .01) than those secreting both metanephrines and normetanephrines (Table 2).

**Table 2.** Relationship between serum inflammation-based scores and demographic, clinical, and pathological characteristics of patients with pheochromocytoma

	NLR	*P* value	PLR	*P* value	LMR	*P* value	SII	*P* value	PNI	*P* value
**Gender**										
Male (n = 24)	2.5 (1.8-3.9)	.85	170.9 (131.7-221.8)	.58	2.9 (2.3-3.4)	.51	741.6 (468.0-942.6)	.93	52.3 (47.6-55.8)	.17
Female (n = 44)	2.3 (1.6-3.8)		164.6 (112.8-230.4)		3.1 (2.3–4.1)		806.1 (376.7-1188)		54.5 (50.0-57.5)	
**Ethnicity (n = 56)**										
Caucasian (n = 46)	2.7 (1.9-4.4)	.**005**	165.8 (124.9-229.6)	.23	2.8 (2.2-3.4)	.**001**	787.5 (449.3-1145.0)	.12	53.2 (49.8-57.1)	.37
Non-Caucasian (n = 10)	1.6 (1-2.5)		131.2 (83.3-221.3)		4.6 (3.3-5.9)		616.5 (275.2-945.1)		55.3 (50.4-58.4)	
**Symptoms of pheochromocytoma**										
Yes (n = 31)	2.3 (1.7-3.3)	.37	164.6 (112.4-222.0)	.28	3.1 (2.5-3.8)	.29	742.9 (375.0-1167.0)	.57	56 (51.5-58.0)	.05
No (n = 37)	2.6 (1.8-4.2)		170.6 (133.3-247.5)		2.9 (2.2-3.6)		806.1 (515.3-958.4)		52.5 (47.8-55.0)	
**Incidental mass**										
Yes (n = 32)	2.4 (1.8-4.2)	.73	174.3 (131.8-260.9)	.15	2.9 (2.1-3.4)	.24	813.8 (511.2-1172.0)	.40	51.5 (46.8-55.8)	.06
No (n = 36)	2.5 (1.7-3.6)		159.8 (112.6-217.7)		3.1 (2.5-3.8)		738.7 (376.0-1133.0)		54.8 (51.6-57.5)	
**Germline mutations**										
Negative (n = 28)	2.2 (1.8-2.9)	.16	159.6 (124.6-210.4)	.97	2.8 (2.3-3.4)	.46	656.1 (388.2-897.3)	.33	53.5 (49.3-59.1)	.26
Positive (n = 14)	2.1 (1.9-4.6)		166.9 (99.5-207.3)		3.2 (2.2-4.3)		870.2 (407.9-1703)		55.5 (53.3-58.6)	
**CVE**										
Yes (n = 13)	2.6 (1.8-4.8)	.55	221.1 (149.9-258.6)	.17	2.8 (2-3.8)	.59	837.5 (583.8-1349.0)	.48	51.5 (46.3-56)	.12
No (n = 55)	2.4 (1.8-3.3)		154.9 (118.0-222.0)		3 (2.4-3.8)		734.5 (399.1-973.6)		54 (50.0-57.5)	
**HTN**										
Yes (n = 52)	2.7 (1.8-4.2)	.11	176.7 (119.6-240.9)	.15	3 (2.3-3.8)	.85	821.4 (468.0-1342.0)	.07	53 (49.3-57.5)	.58
No (n = 16)	2 (1.6-2.8)		138.7 (124.4-176.6)		2.9 (2.5-3.8)		543.3 (384.2-894.0)		54.5 (51.8-56.0)	
**T2DM**										
Yes (n = 17)	2.8 (1.5-3.8)	.95	169.1 (117.6-246.7)	.72	3.2 (2.4-4.7)	.31	845.7 (502.5-1399.0)	.57	53.5 (51.0-56.9)	.68
No (n = 32)	2.3 (1.8-3.7)		176.7 (125.9-249.3)		3 (2.2-3.6)		716.1 (468.0-1154.0)		52.5 (47.9-57.9)	
**Obesity**										
BMI <30 (n = 46)	2.6 (1.9-4.6)	.**02**	186.1 (138.5-242.2)	.**0005**	2.9 (2.2-3.8)	.**04**	874.7 (562.9-1393.0)	.**0009**	52.5 (48.0-57.0)	.17
BMI ≥30 (n = 19)	2 (1.4-2.9)		124.5 (82.8-173.9)		3.3 (2.7-4.7)		456 (329.3-833.5)		55 (50.8-58.3)	
**Catecholamine excess**										
Both MN-NMN (n = 40)	136 (106.2-168)	*^a^*.03	518.9 (493.5-548.8)	*^a^*.41	130.4 (115.7-144.6)	*^a^*.03	672.1 (634.5-720.5)	*^a^*.43	396.6 (387.4-402.3)	*^a^*.01
NMN (n = 19)	109.9 (92.4-132.6)	*^b^*.99	493.8 (449-540.7)	*^b^*.99	148.2 (134.6-179.2)	*^b^* 0.99	638.2 (589.3-701.8)	*^b^*.99	406.9 (396.1-411.9)	*^b^*.99
MN (n = 7)	115.5 (97.3-172.9)	*^c^*.99	497.7 (461.5-517.3)	*^c^*.42	155.8 (116.3-207.9)	*^c^*.22	643 (554.7-679.8)	*^c^*.30	402.5 (398-406.9)	*^c^*.64
**Median mass size**										
<4.9 cm (n = 34)	2.4 (1.8-1.7)	.65	166.4 (124.0-224.0)	.99	2.8 (2.3-3.8)	.52	742.9 (471.1-939.8)	.64	52.5 (47.3-55.9)	.28
≥4.9 cm (n = 34)	2.6 (1.7-4.0)		160.1 (124.3-237.4)		3.2 (2.5-3.7)		787.5 (387.3-1355.0)		54.3 (50.4-57.0)	
**PASS**										
PASS ≤4 (n = 25)	2.3 (1.7-4.2)	.77	136.7 (94.7-230.4)	.18	3.2 (2.3-4.1)	.41	562.9 (360.8-1153.0)	.09	55 (50.8-58.0)	.61
PASS >4 (n = 36)	2.6 (1.7-4.1)		170.3 (138.0-221.8)		2.9 (2.3-3.6)		848.3 (586.5-1208.0)		53.3 (50.1-57.0)	

Continuous variables are reported as median (IQR) and statistical analysis were performed by 2-tailed Mann-Whitney *U* test.

Abbreviations: BMI, body mass index; CVE, cardiovascular events; HTN, hypertension, T2DM, type 2 diabetes mellitus; LMR, lymphocytes-to-monocytes ratio; MN, metanephrine; NLR, neutrophil-to-lymphocyte ratio; NMN, normetanephrine; PASS, Pheochromocytoma of the Adrenal Gland Scaled Score; pheo, pheochromocytoma; PLR, platelet-to-lymphocyte ratio; PNI, prognostic nutrition index; SII, systemic immune-inflammation index.

^*a*^NMN vs both MN-NMN.

^*b*^NMN vs MN.

^*c*^MN vs both MN-NMN.

### Hematological Parameters in Patients With Pheochromocytoma, NFAT and EAH

**Figure 1.**
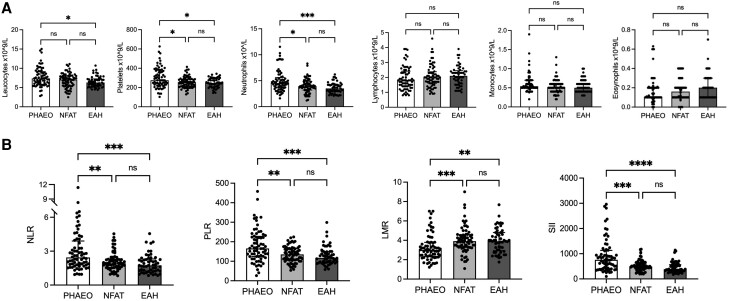
Full blood count and inflammation-based scores of patients with pheochromocytoma (PHEO, n = 68), nonfunctioning adrenal tumor (NFAT, n = 68), and essential hypertension (EAH, n = 53). Comparison of full blood count (A) and inflammation-based scores evaluated (B) between patients with pheochromocytoma and 2 control groups. Data are reported as median and interquartile range, and the upper and the lower whiskers represent, respectively, the 90th and the 10th percentiles. Statistical analysis was performed by Kruskal-Wallis test followed by Dunn post hoc test (*P < .03; **P < .002; ***P value < .0001) Abbreviations: LMR, lymphocytes-to-monocytes ratio; NLR, neutrophil-to-lymphocyte ratio; PLR, platelet-to-lymphocyte ratio; PNI, prognostic nutrition index; SII, systemic immune-inflammation index.

Patients with pheochromocytoma had a higher prevalence of pro-inflammatory changes compared with the NFAT and EAH groups, whereas no differences were found between NFAT and EAH (Fig. 1). Patients with pheochromocytoma had higher leukocyte levels than EAH (median 7.4 [5.9-9.2] vs 6.3 [5.3-7.3]; *P* = .01), mainly because of an increased neutrophil count (median 4.6 [3.6-5.6] vs 3.5 [2.9-4.3]; *P* = .0002). Neutrophil counts were also higher than in NFAT (4 [3.2-4.9]; *P* = .04). In addition, patients with pheochromocytoma had a higher platelet count (median 275 [227.3-378.5]) than those with NFAT (255 [208.5-289], *P* = .04) and EAH (247 [208.5-291.5]; *P* = .01). No significant differences were found in the lymphocyte, monocyte, and eosinophil counts.

Focusing on inflammation-based scores, NLR and PLR were higher in patients with pheochromocytoma (median, 2.4 [1.8-3.9] and 165.8 [124.3-227.5], respectively) and decreased in patients with NFAT (median NLR, 1.9 [1.6-2.3], *P* = .009, and median PLR 135 [105.8-165.6], *P* = .005) and EAH (median NLR 1.7 [1.2-2.3], *P* = .0001, and median PLR 117.7 [94.2-150.2], *P* = .0002). The same trend was observed for SII (pheochromocytoma median, 758.8 [416.1-1133] vs NFAT median, 489.3 [396.4-630], *P* = .0006 vs EAH median 385 [312.7-564], *P* < .0001). Moreover, LMR values were lower in patients with pheochromocytoma (3 [2.3-3.8]) than in those with NFAT and EAH (3.9 [3.3-4.6], *P* = .0003 and 3.9 [2.9-4.8], *P* = .002, respectively) (Fig. 1B).

### Relationship Between Hematological Parameters and Metanephrine Levels and With CRP

**Figure 2.**
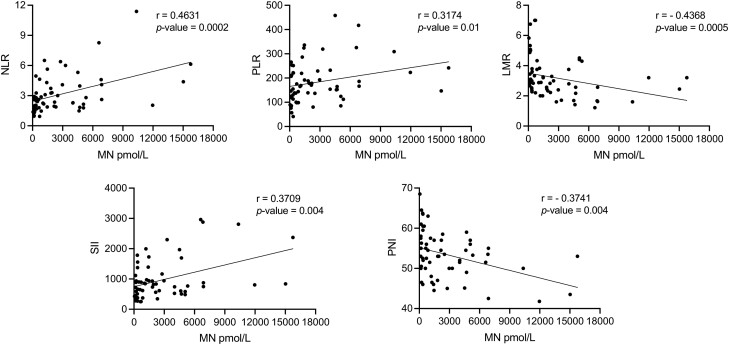
Correlation between inflammation-based scores with metanephrine levels in patients with pheochromocytoma at the time of diagnosis (n = 59). Correlation analysis was performed by Spearman test. Abbreviations: LMR, lymphocytes-to-monocytes ratio; MN, metanephrine; NLR, neutrophil-to-lymphocyte ratio; PLR, platelet-to-lymphocyte ratio; PNI, prognostic nutrition index; SII, systemic immune-inflammation index.

Plasma metanephrine levels significantly correlated with all the assessed inflammation-based scores (Fig. 2). In particular, NLR (*r* = +0.463, *P* = .0002), PLR (*r* = +0.317, *P* = .01), and SII (*r* = +0.371, *P* = .004) correlated positively, whereas LMR (*r* = −0.437, *P* = .0005) and PNI (*r* = −0.374, *P* = .004) correlated negatively. Apart from PLR, in which the association did not reach statistical significance (*P* = .054), the correlations were confirmed after adjustment for age, sex, ethnicity, BMI, and tumor size of pheochromocytoma (Supplementary Table S3) (22). In contrast, normetanephrine levels did not correlate with inflammation-based scores, whereas a significant positive correlation with tumor size was found (*r* = +0.643, *P* = <.0001, Supplementary Fig. S2) (22).

Similarly, CRP was not associated with any of the inflammation-related scores evaluated (NLR *P* = .82; PLR *P* = .71; LMR *P* = .94; SII *P* = .74; PNI *P* = .25).

### Hematological Parameters in Patients With Pheochromocytoma After Adrenalectomy

**Figure 3.**
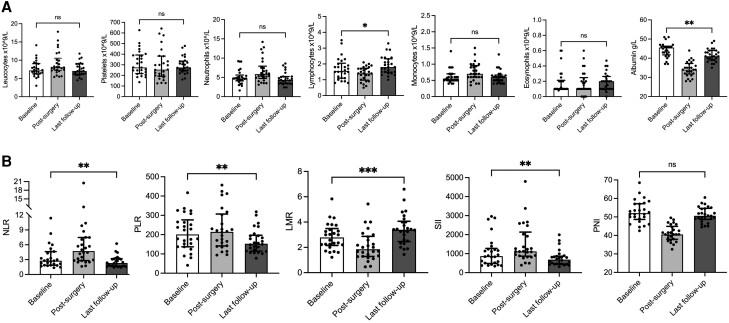
Postoperative changes in complete blood count and inflammation-based scores in patients with pheochromocytoma. Changes in complete blood count (A) and inflammation-based scores (B) at the time of tumor diagnosis (baseline) and at 2 different times after adrenalectomy: on the last day of hospitalization (postsurgery, n = 68) and more than 1 month after surgery (last follow-up, n = 26). Statistical analysis was performed by Wilcoxon signed-rank test. Data are reported as median and interquartile range. Abbreviations: LMR, lymphocytes-to-monocytes ratio; NLR, neutrophil-to-lymphocyte ratio; PLR, platelet-to-lymphocyte ratio; PNI, prognostic nutrition index; SII, systemic immune-inflammation index.

Inflammatory parameters showed a significant improvement after more than 1 month of surgery and the subsequent resolution of catecholamine excess (median time, 15.6 [5.7-42.3] months). Indeed, the lymphocyte count increased compared with baseline (*P* = .01), with corresponding changes in the relative inflammation-based scores (Fig. 3B). Specifically, the postoperative NLR was significantly lower than the preoperatively (median values from 2.78 to 2.30, *P* = .001). The median PLR and SII also decreased from 201.2 to 152.7 (*P* = .003) and from 870.2 to 687.3 (*P* = .004), respectively. In addition, LMR increased from diagnosis (median values from 2.79 to 3.39, *P* = .0002). No difference was detected for other parameters of CBC (ie, leukocyte, platelet, neutrophil, monocyte, eosinophil counts) and PNI (*P* = .14, Fig. 3A).

Looking at individual time points, the reduction in systemic inflammation showed a gradual progression after a transient increase immediately after surgery (median, 3 [2-5] days). In detail, an increase in neutrophil and monocyte counts and a decrease in lymphocyte counts and serum albumin levels were observed at the first postoperative assessment (Supplementary Fig. S3A) (22). This led to an increase in NLR (*P* = <.0001) and SII (*P* = .01) and a decrease in MRL (*P* = <.0001) and PNI (*P* = <.0001) compared with the baseline (Supplementary Fig. S3B) (22). Instead, as shown in Supplementary Fig. S4 (22), there was a significant reduction in NLR, PLR, and SII (*P* = .0005, .003, and .006, respectively) during the first year after adrenalectomy (median time, 5 months, IQR 1.8-7.9), which tended to decrease further at long-term follow-up (median time, 39.6 months, IQR 26.4-55.2). In addition, the LMR showed a consistent increase over time (*P* = .03 and .02 at short-term and long-term follow-up, respectively), whereas a decrease in PNI was only observed at 1 year after surgery (*P* = .047).

### Influence of α-blocker Therapy on the Hematological Parameters

**Figure 4.**
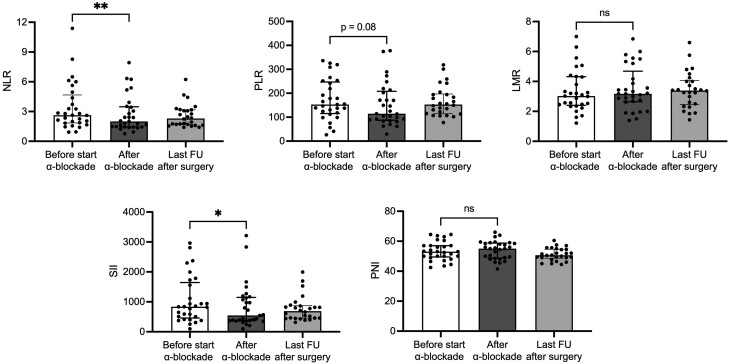
Changes in inflammation-based scores in patients with pheochromocytoma treated with preoperative α-blockade (n = 29). The changes in inflammation-based scores observed after α-blockade treatment were similar to those found in patients evaluated at least 1 month after surgery (last follow-up after surgery). Data are reported as median and interquartile range, and the upper and the lower whiskers represent respectively the 90th and the 10th percentiles. Statistical analysis was performed by Wilcoxon signed-rank test (*P value < .05, **P value < .01). Abbreviations: LMR, lymphocytes-to-monocytes ratio; NLR, neutrophil-to-lymphocyte ratio; PLR, platelet-to-lymphocyte ratio; PNI, prognostic nutrition index; SII, systemic immune-inflammation index.

A subcohort of 29 patients with pheochromocytoma was assessed before and after treatment with preoperative α-blockade (Fig. 4). All patients were treated with doxazosin for a median of 110 (IQR 78.5-261.5) days at a median total daily dose of 4 (IQR 2-11) mg. The changes in hematological parameters observed after doxazosin treatment were similar to those found in patients evaluated at least 1 month after surgery (Fig. 4). In fact, lymphocytes were increased (*P* = .01) and neutrophils decreased (*P* = .03), resulting in a significant reduction in the NLR, with median values decreasing from 2.63 to 2 (*P* = .007). As a result, SII, which is dependent on NLR, was also significantly reduced (*P* = .03). Furthermore, PLR values tended to be reduced after the introduction of α-blockade (*P* = .08), whereas LMR and PNI were not different.

## Discussion

Hereby, we provide the first comprehensive study of the relationship between inflammation-based scores, as surrogates for systemic inflammation, and metanephrine levels in patients with pheochromocytoma compared with patients with NFAT or EAH. We studied a cohort of well-characterized patients with pheochromocytoma and, after confirming the presence of a preoperative systemic inflammatory state, we demonstrated the presence of a significant correlation between baseline plasma metanephrine levels and the inflammation-based scores. Moreover, we showed a positive effect of either the removal of the pheochromocytoma or the administration of α-blockers drugs on these scores.

Previous studies have shown that patients with pheochromocytoma are characterized by the presence of pro-inflammatory changes. A review of almost 100 patients with catecholamine-secreting tumors showed that leukocytosis and neutrophilia were a relatively common finding (15). Furthermore, other studies have found that not only CBC parameters but also acute-phase proteins, such as elevated C-reactive protein, were higher in patients with pheochromocytoma than healthy subjects or patients with other types of hypertensive conditions (9, 14, 28, 29).

Our study is the first 1 assessing in a cohort of patients with pheochromocytoma and with the inclusion of control groups the levels of the systemic inflammation in patients using inflammation-based scores, markers proven to reflect inflammatory state in several diseases (17-19, 21). Indeed, the previous study by Van der Heijden and coauthors suggested that patients with pheochromocytoma had higher levels of NLR and monocyte/lymphocyte ratio compared with EAH patients, but the study was performed on only 10 patients (9). On the other hand, Zhong et al analyzed a large series of 728 patients with catecholamine-secreting tumors, but without comparison with any control groups, and investigating only the prognostic role of inflammation-based scores (16). At variance with those previous studies, we performed a comprehensive analysis of 68 patients with pheochromocytoma by assessing several inflammation-based scores and including a comparison with 2 control groups (ie, 53 patients with EAH and 68 patients with NFAT). That significant changes in several inflammatory parameters were observed when comparing patients with pheochromocytoma with these 2 control groups, is of utmost importance. Indeed, as increased catecholamines are a hallmark of pheochromocytoma, the presence of an enhanced inflammatory state and inflammation-based scores, particularly the NLR (30-32), observed in these patients suggests that these hormones may have a greater impact on the systemic inflammatory response than hypertension or the presence of a tumor mass (33-35). This idea is supported by the observation that in our cohort of pheochromocytoma hypertension, CVE events, and T2DM did not significantly affect the inflammation-based scores.

As far as the clinical characteristics possibly influencing the inflammation-based scores in pheochromocytoma is concerned, we observed relationships between these parameters and both ethnicity and body weight. Specifically, Caucasian patients had more inflammatory changes, reflected by higher NLR and lower LMR, than other ethnicities, consistent with previous observations in healthy subjects (36, 37). Therefore, ethnicity may influence the clinical application of the present findings in the management of patients with pheochromocytoma, but the sample sizes analyzed are not large enough to confirm this and further larger studies are required.

Interestingly, contrary to the conventional notion that obesity is associated with chronic inflammation (38, 39), in our study patients with BMI <30 kg/m^2^ showed a more pronounced systemic inflammatory state, as reflected by higher NLR, PLR, and SII and low LMR values, than patients with obesity. In support of this finding, we observed that patients with a lower BMI tended to have higher levels of catecholamine, which are known to increase metabolic rate and induce weight loss (4, 5, 40). Indeed, in our cohort of pheochromocytoma, patients with a BMI <30 kg/m^2^ tended to have higher catecholamine levels than obese patients, although no significant difference was found, probably because of the limited number of patients. Thus, it is not possible to exclude that the apparently surprising more pronounced inflammatory state in patients with BMI <30 kg/m^2^ could be due, in fact, to their tendentially higher catecholamine secretion, which seems to be associate with the inflammation-based scores.

Our study, indeed, is the first to demonstrate that plasma metanephrine levels are significantly correlated with all inflammation-based scores evaluated. In fact, we noticed a direct relationship, with increasing plasma metanephrine levels corresponding to increased NLR, PLR, and SII, with concomitant decreases in LMR and PNI. Therefore, this finding suggests that catecholamines play a direct role in the systemic inflammation in pheochromocytoma, which is further supported by the lack of association found between scores and CRP levels. In particular, metanephrines seem to be more involved than normetanephrine, as evidenced by the lower inflammatory state in patients with pheochromocytomas secreting only normetanephrines. However, the possible different relationship between metanephrine or normetanephrine and inflammation has been poorly investigated and data reported in the literature are discordant. Overall, it seems that the presence of comorbidities (ie, T2DM, insulin resistance, periodontitis, and obstructive sleep apnea syndrome) may play a role in influencing the relationship between metanephrine and inflammation (41, 42).

The postoperative changes observed in the present study further support this hypothesis. The increase in both CBC and inflammation-based scores in the immediate postoperative period may be attributed to an acute stress response induced by the surgical procedure (43). However, we detected a significant reduction in inflammation during the long-term postsurgical monitoring, as also shown by other authors (9, 14, 16). In our study, these changes were more pronounced in the inflammation-based scores than CBC. In fact, at least 1 month after surgery, our patients showed a significant decrease in the lymphocyte count—and consequently NLR, PLR, and SII increased and LMR decreased—suggesting a prevalent impact of lymphocyte compared to monocytes, platelets, and neutrophils in this scenario. In summary, the resolution of changes in inflammation-based scores during long-term follow-up suggests an improvement in the inflammatory status of patients, with a potential benefit for their prognosis. This finding is in keeping with previous data showing that the postoperative reduction of NLR in patients with pheochromocytoma was associated with overall survival (16) and that the inflammation may play a role in the cardiovascular risk of patients with pheochromocytoma (9, 14, 44).

Finally, we investigated whether treatment with α-blockers could influence the preoperative inflammation-based scores. So far, studies in vitro have shown that stimulation of α-adrenergic receptors promotes the production of pro-inflammatory cytokines (7), which is inhibited by α-adrenoceptor antagonists (45-47). Our study is the first to evaluate the influence of α-blockade on inflammation-based scores in patients with pheochromocytoma. In a subset of 29 patients, we observed a reduction in NLR and SII with α-blocker treatment and assumed a favorable influence on the inflammatory state of patients. However, we acknowledge that larger studies are needed to confirm these findings.

We recognize that our study has some limitations. First, because of its retrospective design, we cannot definitively establish a causal relationship between plasma metanephrine levels and inflammation-based scores, despite the observed association. Second, although our study extended the analysis by including several inflammation-based scores compared with previous studies, we did not evaluate more specific inflammatory markers, such as circulating cytokines, interleukins, or acute-phase proteins. Measurement of these markers would provide a more complete assessment of the systemic inflammatory state in patients with pheochromocytoma. Furthermore, we did not have data on plasma chromogranin A, which could be a useful marker to associate with metanephrine levels. However, the sensitivity and specificity of this test are highly variable between studies, so its clinical use in pheochromocytoma remains an open issue (48). Third, in our analysis, we used a single blood count to calculate inflammation-based scores. Although we excluded any possible conditions that might have interfered with the test at the time of sampling, it would have been useful to have multiple measurements available to average for greater accuracy. Finally, the difference in tumor mass size between pheochromocytomas and NFAT may have affected the results of comparing these 2 groups. In fact, we found that pheochromocytoma were significantly larger than NFAT, and it is known that the tumor microenvironment can influence systemic inflammation (34, 35). However, on univariate and multivariate analysis, we observed that the association between metanephrine levels and most inflammation-based scores was maintained even after adjustment for tumor size.

In conclusion, the association between plasma metanephrine levels and preoperative systemic inflammatory status, reflected by high NLR, PLR, and SII as well as low LMR and PNI, that resolves during long-term follow up, suggests that the pro-inflammatory changes in pheochromocytomas are likely related to excessive amount of secreted catecholamines. The impact of circulating catecholamines on the systemic inflammatory response may play a role in the cardiometabolic comorbidities in patients with pheochromocytoma. Understanding the connection between catecholamine levels, inflammation, and comorbidities may optimize treatment approaches, potentially improving outcomes and quality of life for individuals with pheochromocytoma. Further research is needed to confirm the exact mechanism by which catecholamines influence the systemic inflammation.

## Data Availability

All the relevant data underlying this article are available in the article and in its online supplementary material (22). Additional data will be shared on reasonable request to the corresponding author.

## Abbreviations

BMI: body mass index
CBC: complete blood count
CRP: C-reactive protein
CVE: cardiovascular events
EAH: essential hypertension
IQR: interquartile range
LMR: lymphocytes-to-monocytes ratio
NFAT: nonfunctioning adrenocortical tumor
NLR: neutrophil-to-lymphocyte ratio
PASS: Pheochromocytoma of the Adrenal Gland Scaled Score
PLR: platelet-to-lymphocyte ratio
PNI: prognostic nutrition index
SII: systemic immune-inflammation index
T2DM: type 2 diabetes mellitus
